# A Method for the Prediction of Clinical Outcome Using Diffusion Magnetic Resonance Imaging: Application on Parkinson’s Disease

**DOI:** 10.3390/jcm9030647

**Published:** 2020-02-28

**Authors:** Chih-Chien Tsai, Yu-Chun Lin, Shu-Hang Ng, Yao-Liang Chen, Jur-Shan Cheng, Chin-Song Lu, Yi-Hsin Weng, Sung-Han Lin, Po-Yuan Chen, Yi-Ming Wu, Jiun-Jie Wang

**Affiliations:** 1 Healthy Aging Research Center, Chang Gung University, Taoyuan 33302, Taiwan; nasnas218@mail.cgu.edu.tw; 2 Department of Medical Imaging and Intervention, Chang Gung Memorial Hospital, Linkou, Taoyuan 33375, Taiwan; jack805@gmail.com (Y.-C.L.); shng@adm.cgmh.org.tw (S.-H.N.); chenyl0702@adm.cgmh.org.tw (Y.-L.C.); jimmy_cgmh@hotmail.com (Y.-M.W.); 3 Department of Medical Imaging and Radiological Sciences, Chang Gung University, Taoyuan 33302, Taiwan; image.lin@gmail.com (S.-H.L.); lmrrra3@gmail.com (P.-Y.C.); 4 Department of Diagnostic Radiology, Chang Gung Memorial Hospital, Keelung City 20401, Taiwan; 5 Clinical Informatics and Medical Statistics Research Center, College of Medicine, Chang Gung University, Taoyuan 33302, Taiwan; jscheng@mail.cgu.edu.tw; 6 Department of Emergency Medicine, Chang Gung Memorial Hospital, Keelung City 20401, Taiwan; 7 Professor Lu Neurological Clinic, Taoyuan 33375, Taiwan; c81214@adm.cgmh.org.tw; 8 Division of Movement Disorders, Department of Neurology, Chang Gung Memorial Hospital, Linkou, Taoyuan 33375, Taiwan; yhweng@adm.cgmh.org.tw; 9 Neuroscience Research Center, Chang Gung Memorial Hospital, Linkou, Taoyuan 33375, Taiwan; 10 School of Medicine, Chang Gung University, Taoyuan 33302, Taiwan; 11 Medical Imaging Research Center, Institute for Radiological Research, Chang Gung University/Chang Gung Memorial Hospital, Linkou 33375, Taoyuan, Taiwan

**Keywords:** diffusion tensor imaging, least absolute shrinkage and selection operator, machine learning, Parkinson’s disease, prognosis

## Abstract

Robust early prediction of clinical outcomes in Parkinson’s disease (PD) is paramount for implementing appropriate management interventions. We propose a method that uses the baseline MRI, measuring diffusion parameters from multiple parcellated brain regions, to predict the 2-year clinical outcome in Parkinson’s disease. Diffusion tensor imaging was obtained from 82 patients (males/females = 45/37, mean age: 60.9 ± 7.3 years, baseline and after 23.7 ± 0.7 months) using a 3T MR scanner, which was normalized and parcellated according to the Automated Anatomical Labelling template. All patients were diagnosed with probable Parkinson’s disease by the National Institute of Neurological Disorders and Stroke criteria. Clinical outcome was graded using disease severity (Unified Parkinson’s Disease Rating Scale and Modified Hoehn and Yahr staging), drug administration (levodopa equivalent daily dose), and quality of life (39-item PD Questionnaire). Selection and regularization of diffusion parameters, the mean diffusivity and fractional anisotropy, were performed using least absolute shrinkage and selection operator (LASSO) between baseline diffusion index and clinical outcome over 2 years. Identified features were entered into a stepwise multivariate regression model, followed by a leave-one-out/5-fold cross validation and additional blind validation using an independent dataset. The predicted Unified Parkinson’s Disease Rating Scale for each individual was consistent with the observed values at blind validation (adjusted R^2^ 0.76) by using 13 features, such as mean diffusivity in lingual, nodule lobule of cerebellum vermis and fractional anisotropy in rolandic operculum, and quadrangular lobule of cerebellum. We conclude that baseline diffusion MRI is potentially capable of predicting 2-year clinical outcomes in patients with Parkinson’s disease on an individual basis.

## 1. Introduction

Parkinson’s disease (PD) is a common progressive neurodegenerative disorder characterized by resting tremor, bradykinesia, restricted mobility, and postural instability. The diagnosis of PD relies primarily on clinical signs and symptoms according to commonly accepted criteria [1]. PD has a progressive course [2] and is associated with increased mortality [3], with physical disabilities and non-motor symptoms exerting a significant negative impact on the quality of life [4]. In this context, robust early prediction of clinical outcomes would be paramount for implementing appropriate interventions. Unfortunately, reliable biomarkers of clinical outcome and/or progression are still lacking. Besides, the assessment of clinical outcomes can be time-consuming and might fluctuate according to the patient’s conditions at the time of measurement.

Magnetic resonance imaging (MRI) is a medical imaging technique that provides excellent tissue contrast without using ionizing radiation. Although patients with advanced neurodegenerative disease may show signs of cerebral atrophy [5], the use of MRI in suspected patients is generally restricted to rule out concomitant brain disorders, rather than for diagnostic confirmation. The application in prognosis is even further limited to no or weak correlation with clinical outcomes, for example, disease severity, drug administration, and quality of life [6,7].

Diffusion MRI has been utilized to investigate microstructural damage in the brain of patients with PD. For example, the free-water level in the posterior substantia nigra, as measured by diffusion MRI, could potentially predict the changes in bradykinesia and cognitive status over one year [8,9]. A recent study using diffusion tensor imaging (DTI) showed that extensive cortical regions were altered in patients with PD when compared to age-matched controls [10]. The identification of these changes has important diagnostic implications. Such extensive cortical involvement in PD might lead to impairments in the related sensorimotor or cognitive functions and, as a consequence, contribute to the clinical outcome [11]. Our hypothesis is that the clinical outcome can be predicted by a comprehensive assessment of the pattern in the entire brain, rather than a few etiology-related regions.

To select multiple involved regions, conventional analysis based on the manual delineation of regions of interest can be time-consuming and subjective. It would be impractical to select many regions of interest throughout the brain, especially when multiple slices are needed to identify a 3D structure. A representative slice could be used, but might not necessarily be located where the disease-related signal alteration has occurred. The use of voxel-wise analysis may serve as a potential alternative, but either fails to account for inter-individual variation (in the absence of normalization) or alters the principal diffusion direction (when normalization is applied). Brain parcellation with standard template after normalization to obtain multiple parcellated regions would allow diffusion parameters from standardized brain regions to be obtained, and therefore makes multivariate regression model analysis possible.

Although the whole brain parcellation approach allows for the identification of many potentially neglected brain regions, challenges against the traditional statistical approach due to high feature dimensionality still occur, which could lead to overfitting of the data [12]. To avoid the potential spurious correlation, we propose to perform feature reduction by least absolute shrinkage and selection operator (LASSO), followed by a general linear regression model in our bivariate associations. This procedure has been shown to be stable for predictive performance [13].

As such, diffusion measurements from the parcellated brain as a whole was reduced by LASSO to improve the generalization of the established prognostic model. The meaningful features that remained could be helpful in elucidating the relationship between underlying microstructural damage and clinical outcome. We therefore designed the current longitudinal study to investigate whether baseline diffusion parameters can predict clinical outcomes in patients, using PD as an example. Specifically, our purpose is to examine the prognostic performance of diffusion parameters in the prediction of disease severity, drug administration, and quality of life in patients with PD. If confirmed, our study would demonstrate the possibility of diffusion parameters, as measured from parcellated multiple brain regions, to predict the clinical outcomes of patients with PD after 2 years.

## 2. Material and Methods

This prospective study was approved by the local Institutional Review Board (100-3761A3). After a detailed explanation of the study, all participants provided written informed consent.

### 2.1. Patients

A total of 109 patients with PD (61 males and 48 females, mean age: 61.2 ± 7.1 years) were recruited between June 2012 and May 2013. The cross-sectional analysis of patients in the first visit was previously published in 2016 [10]. Only patients who returned for follow-up examination were included in the analysis. In total, 82 subjects were included (45 males and 37 females, mean age: 60.9 ± 7.3 years), who visited between June 2014 and 2015 (mean follow-up time 23.7 ± 0.7 months). Of the 27 excluded patients, 17 were unwilling to undergo a second visit, 1 expired in an accident, 4 underwent deep brain stimulation, and 5 had poor image quality at baseline. Diagnosis and ad hoc recruitment were performed by the consensus of three senior neurologists (28 years, 21 years, and 8 years of experience). The inclusion criteria were as follows: (1) diagnosis of probable PD, as proposed by the National Institute of Neurological Disorders and Stroke, except for the age at onset [14]; (2) ability to tolerate treatment discontinuation for 12 h; (3) modified Hoehn and Yahr staging (MHY) ratings between 1 and 3 at baseline. Table 1 shows the general characteristics of the participants.

The exclusion criteria were general MRI exclusion criteria as well as the presence of any of the following conditions: (1) major physical illnesses (i.e., renal failure, heart failure, stroke, acute myocardial infarction, unstable angina, poorly controlled diabetes mellitus, poorly controlled hypertension, moderate-to-severe dementia, severe dyskinesia, and cancer), (2) psychiatric disorders, (3) known brain abnormalities, (4) history of intracranial surgery, and (5) pharmacotherapy for more than ten years or treatment with drugs able to cross the blood–brain barrier (other than those used to treat PD).

All participants were diagnosed and treated according to standard routine. They underwent a detailed medical history review and neurological and physical examinations. The following parameters were recorded: body mass index, education, Mini-Mental State Examination (MMSE), Schwab and England Activity of Daily Living (ADL) score, and the Taiwanese version of the Medical Outcomes Study Short Form-36 (SF-36SI) [15]. Patients deemed eligible were scheduled for MRI examinations. The following measures were used in the analysis: clinical outcome according to the disease severity (Unified Parkinson’s Disease Rating Scale, UPDRS; MHY), and levodopa equivalent daily dose (LEDD). Quality of life was assessed using the 39-item PD Questionnaire (PDQ39). The summary indices (PDQ39SI) were used in the final analysis. In addition, images of T_2_-weighted turbo spin-echo, T_2_-weighted fluid attenuation inversion recovery, and T_1_-weighted magnetization-prepared rapid gradient-echo were acquired to rule out the presence of any intracranial structural abnormality. MR images were read independently by one of three neuro-radiologists who were blinded to the diagnosis (27 years, 19 years, and 9 years of experience).

### 2.2. Imaging

MRI was performed with a 3T scanner using a 12-channel head matrix coil (Trio, A TIM system, Magnetom, Siemens, Erlangen, Germany). The patient’s head was kept fixed with a pad to avoid bulk motion during scanning. The imaging parameters for T1-weighted images were repetition time/echo time/inversion time/flip angle = 2000 ms/2.63 ms/900 ms/9°, slice thickness = 1 mm, number of slices = 160, matrix size = 224 × 256, field of view = 224 × 256 mm^2^, and acquisition time = 4 min 8 s.

Diffusion-weighted images were acquired using a spin-echo echo-planar-imaging sequence with repetition time/echo time/slice thickness = 5700 ms/108 ms/3 mm, matrix size = 96 × 96, and field of view = 192 × 192 mm^2^, 40 slices which covered the whole brain down to the cerebellum, and an acceleration factor of 2 using GRAPPA (generalized autocalibrating partially parallel acquisition) reconstruction. The *b*-value was 1000 s/mm^2^ with the diffusion-weighting gradients applied along 30 non-collinear directions.

#### 2.2.1. Image Post-Processing

Diffusion data were processed using Diffusion Kurtosis Estimator software [16]. The mean diffusivity (MD) and fractional anisotropy (FA) were extracted from the diffusion tensor in accordance with the methods described by Lo et al. [17]. Briefly, a transformed matrix was identified by normalizing the B_0_ image from each individual to the standard Montreal Neurological Institute template using linear registration (12 parameter affine transformation) and was parcellated into 116 different regions according to the Automated Anatomical Labelling template [18]. A parenchyma mask was used to remove the presence of cerebrospinal fluid in all diffusion metrics. The 10th, 50th, and 90th percentiles for each parcellated brain region were recorded, which led to 696 features in each subject.

#### 2.2.2. Statistical Analysis

Statistical analyses were performed in SAS version 9.4 (SAS Institute, Inc. Cary, NC, USA). Student’s paired *t*-test was used to examine the differences in (a) clinical measures (*p* < 0.05), and (b) diffusion parameters between baseline and follow-up. The normal distribution of the diffusion index was tested using the Kolmogorov–Smirnov test. Statistical significance was reached at *p* < 0.05 with correction for multiple comparisons by using Bonferroni’s method (0.05/696) in tests for diffusion parameters.

For blind validation, the original data were randomly split into two sub-datasets by using systematic random sampling. Following the 80/20 rule [19,20], the training dataset consisted of 66 patients (80%) and independent blind dataset of 16 (20%). All qualified patients were enrolled in a chronological order and selected at a fixed interval. Regression analysis used LASSO, in which L1-norm regularization was performed to minimize the sum of the absolute value of the regression coefficients [21] in order to reduce the number of features to a statistically reasonable level for further analyses [22]. Age, sex, disease duration and imaging protocols were examined by LASSO, together with the imaging features, in order to clarify the potential confounding effect on the predictability of the models. During the training process, approximately 43 to 55 features, less than the sample size, were selected by LASSO from the original 696 imaging parameters for each clinical change. To devise a final predictive model, a stepwise linear regression was performed from LASSO selected features using 1000 times bootstrap calculation. To avoid overfitting the model, the number of features was further reduced to 1/5 of the training samples [23,24]. As a result, 13 features were entered into the regression model.

Adjusted R^2^, F values, and regression coefficients were used to express goodness-of-fit. Predictive power was expressed as adjusted R^2^. To confirm our final model, we employed a leave-one-out and five-fold cross validation in the training dataset, followed by blind validation in the independent blind dataset, where the mean values of the adjusted R^2^ and the absolute error (MAE, the absolute difference between the observed and the predicted scores) were calculated from each validation model.

**Figure 1:**
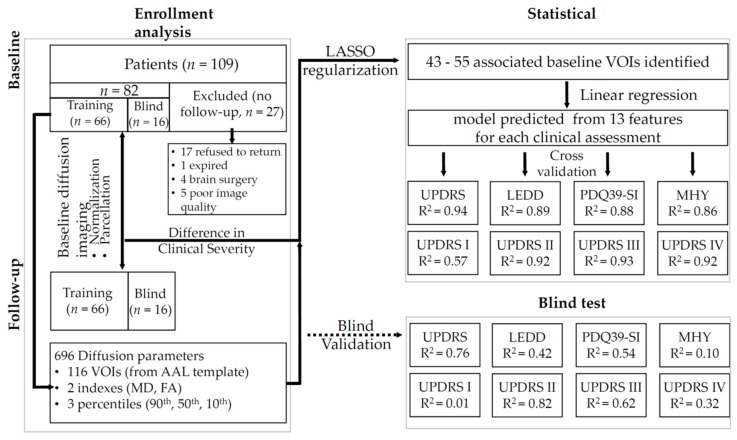
Experimental design from enrollment to statistical analysis. Changes in both structural and diffusion images, as well as clinical outcome measures, including disease severity (UPDRS and MHY), LEDD, and PDQ39, were compared between baseline and follow-up. Baseline diffusion parameters entered statistical analysis. The prognostic performance was examined for the entire cohort, where the adjusted R^2^ for clinical outcome measures were presented.

Figure 1 shows the experimental procedure from enrollment to statistical analysis.

## 3. Results

### 3.1. Changes over the Study Period

We first looked for any changes from baseline to the end of the study in clinical outcome, dose administration, and quality of life (Table 1). None of the patients had an intracranial structural abnormality. Over the 2-year study period, clinical staging (MHY) increased by 0.3 (*p* < 0.001), as did the total and the motor subscale of the UPDRS by 6.1 and 2.5, respectively (*p* = 0.003). In the assessments of quality of life (including in ADL, UPDRS category II, PDQ39SI, and SF36SI), there were significant declinations (*p* < 0.005). Measures of disease severity were not correlated with age, systolic or diastolic blood pressure, education, and body mass index. A significant positive correlation was found between disease duration and clinical outcomes (*p* < 0.05).

**Figure 2:**
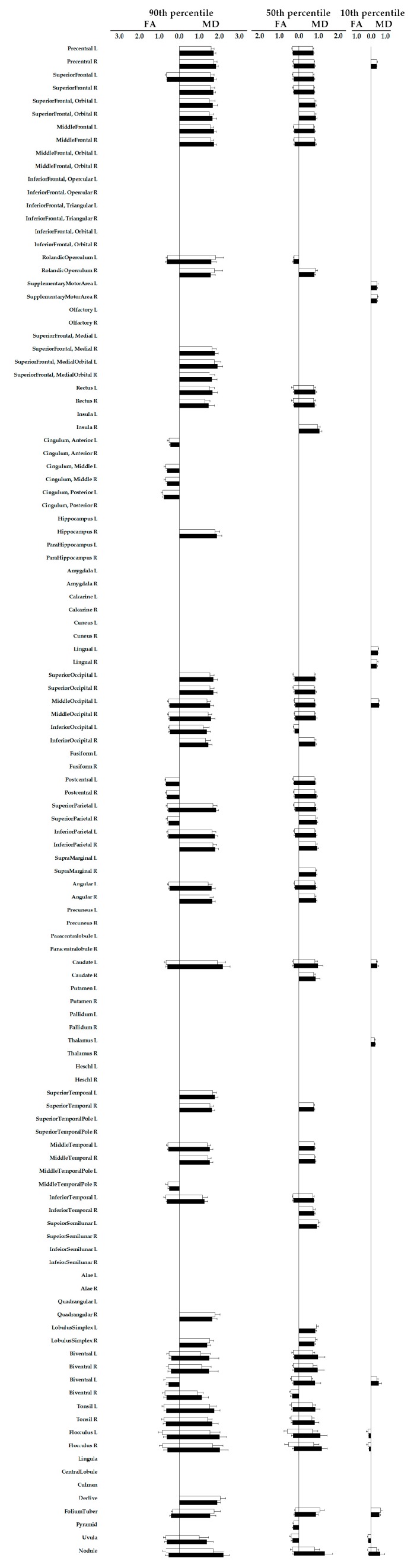
Changes of diffusion parameters from the parcellated brain regions over the follow-up period. The figure shows the change of diffusion parameter from parcellated brain regions in patients with PD if with statistically significant differences between baseline and the end of the study. MD, mean diffusivity, in unit of 10^−3^ mm^2^/sec; FA, fractional anisotropy, unitless.

The values of FA and MD at baseline and follow-up are summarized in Table 2. Figure 2 plots the changes in diffusion parameters over the follow-up period when significant. Extensive regions can be involved, which include superior and middle parts of the frontal lobe; superior, middle and inferior parts of the occipital lobe; biventral, tonsil, and flocculus of the cerebellum. Notably, in basal ganglia, the regions with a significant change in diffusion were mainly located in the caudate.

### 3.2. Summary of Regression Analysis

Imaging parameters that survived the LASSO regression with the change of each clinical outcome in the training dataset were entered into the linear regression analysis. All of the variance inflation factors were within the normal range and <2.8, which suggests that the assumption of multicollinearity was not violated. The values of statistical power and effect size (Cohen f^2^) of the prediction model ranged from 0.94–1 and 0.48–7.59, respectively.

**Figure 3:**
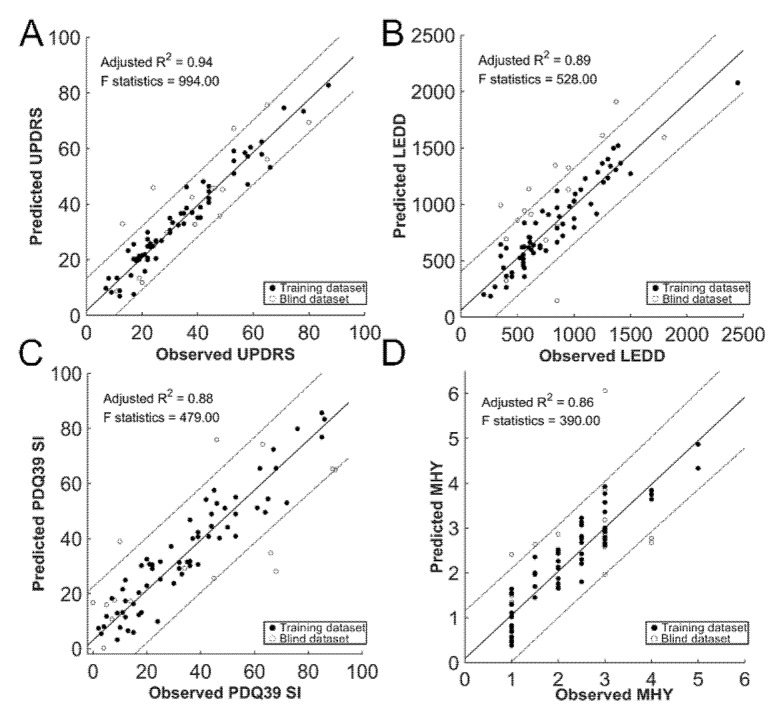
Prediction of clinical outcome measures: regression results. The predicted clinical decay against the observed outcomes on an individual basis, including the total score of UPDRS (panel **A**), levodopa equivalent daily dose (LEDD, panel **B**), Summary Index of 39-item PD Questionnaire (PDQ39SI, panel **C**) and Modified Hoehn and Yahr staging (MHY, panel **D**). Solid, training dataset; circle, blind dataset.

**Figure 4:**
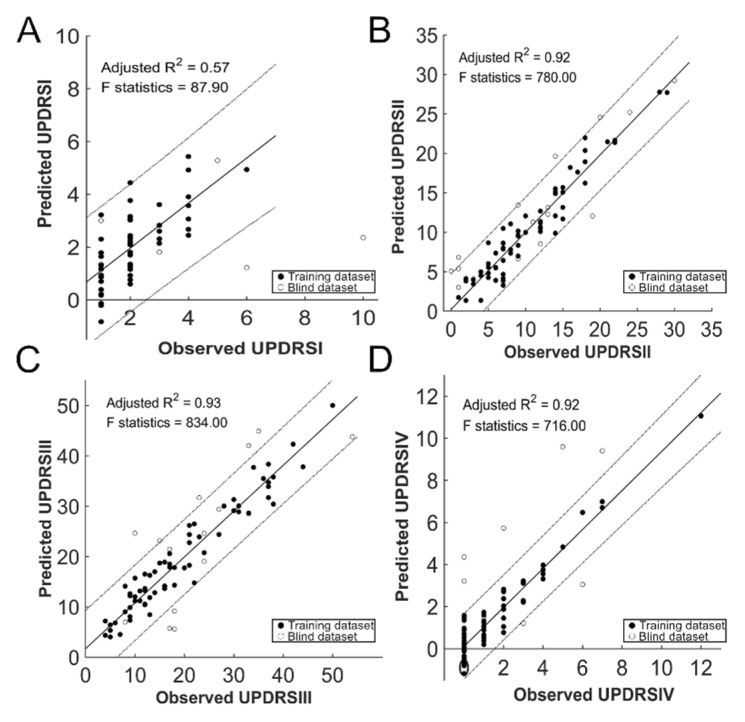
Prediction of Unified Parkinson’s Disease Rating Scale: regression results. The predicted clinical decay of each subscale of the UPDRS (Category I, panel **A**; Category II, panel **B**; Category III, panel **C**; Category IV, panel **D**) on an individual basis against the observed outcomes. Solid, training dataset; circle, blind dataset.

Regression analyses revealed strong-to-excellent (adjusted R^2^ between 0.57 and 0.94) associations between the predicted clinical parameters by baseline diffusion parameters and the observed at follow-up. We summarized the results of the regression, cross- and blind validations, including the adjusted R^2^ and the mean average error in Table 3. The predicted scores at follow-up are plotted against the observed values in Figure 3, including the disease severity (total score of UPDRS, MHY), LEDD, and PDQ39SI, using both the training (solid circle) and the blind (circle) datasets. The observed UPDRS at follow-up were significantly in line with those predicted by the regression model with the high adjusted R^2^ (leave-one out/5-folds cross validation = 0.939 ± 0.002/0.944 ± 0.011, Figure 3A). Figure 4 shows the predicted and observed values for each category in UPDRS in the training dataset (solid circle) and the blind dataset (circle). Category II and III in UPDRS showed highly adjusted R^2^, which ranged between 0.923 and 0.933 in cross validations.

For the independent blind dataset, the adjusted R^2^ was 0.76 in UPDRS with an inherent error of 4.38%. The linear relationships between the predicted and observed LEDD, PDQ39SI, and MHY were also good-to-excellent (adjusted R^2^ between 0.86 and 0.89, Figure 3B–D). Noticeably, LEDD could be predicted at a mean adjusted R^2^ of 0.891 and 0.898 at both cross and blind validations with a mean average error of approximately 135 and 138 mg, respectively (Table 3). The adjusted R^2^ was still high for Category II (0.82) and III (0.62) in UPDRS at the independent blind dataset. The error between the predicted and the observed scores were 5.88% and 6.90%, respectively.

### 3.3. Prediction of the Clinical Outcome

The clinical outcome can be calculated from the equations of linear regression, as listed in Table 4 (UPDRS and each sub-category) and Table 5 (MHY, LEDD, and PDQ39SI). Dependent variables and the corresponding unstandardized coefficients for each clinical outcome are also reported. The clinical changes in individual patients could be calculated by the combination of diffusion parameters calculated from multiple brain regions, which showed that all regression models involved the cerebellum. Notably, both the total score and the motor subscale of UPDRS could be predicted by diffusion parameters measured from several overlapping regions, including the caudate, rectus, and, most pronounced, the cerebellum. In Category II, the predictive regions were most notably the visual cortex, thalamus, insula, cingulum, hippocampus, and cerebellum.

In the assessment of life quality (PDQ39SI), the adjusted R^2^ was 0.88 at both validations, mostly predicted by diffusion indices measured in the occipital lobe, cingulum gyrus, quadrangular and biventral lobules in the cerebellum, rolandic, insula, caudate, paracentral gyrus, temporal, frontal lobe, and lingula of cerebellum vermis. Regions that entered into the regression model of LEDD were located in the paracentral gyrus, frontal lobe, Heschl gyrus, postcentral, occipital lobe, cuneus, thalamus, temporal lobe, precuneus, and parahippocampal gyrus, alae, biventral, and tonsil lobules of cerebellum.

## 4. Discussion

### 4.1. Major Findings and Clinical Impacts

The study proposes a multivariate approach to examine the potential prognostic performance of diffusion MRI on later disease severity, LEDD, and PDQ-39 of patients with PD. The results of our study indicate that the clinical outcome over a 2-year follow-up period can be confidently predicted by baseline diffusion parameters measured in parcellated regions from the whole brain. Importantly, diffusion parameters were found to predict the clinical trajectory of PD over time on an individual patient basis. The results were blind-validated by using an independent sub-dataset, establishing the validity of prognostic prediction by features from diffusion MRI.

Diffusion imaging can be easily incorporated into routine MRI examination, which has often been prescribed in patients suspected of having PD to rule out concomitant brain disorders. Here, we show the prognostic value of our approach by using different diffusion parameters in patients with PD. The proposed machine learning algorithm might help facilitate the development of an artificial intelligence-based computer-aided prognosis in patients with PD using diffusion tensor imaging. Diffusion tensor imaging can be objective and does not require extensive cooperation from the patient [25]. Our study demonstrates the value of adding this step to routine imaging examinations. This might significantly contribute to the confidence of neurologists on the diagnosis and prognosis of PD.

### 4.2. Prediction of Motor Function

Owing to their paramount pathogenetic role in PD, the substantia nigra, in particular the basal ganglia, has been the subject of intense MRI investigation [26,27,28,29]. However, the detection of structural changes in the middle temporal gyrus more than in the basal ganglia may improve diagnostic accuracy [10,30]. Our study shows that during the follow-up period, changes in diffusivity can occur in extensive cortical regions, for example, middle frontal gyrus, superior and middle occipital gyri, as well as nodule and tonsil in the cerebellum. Because microenvironmental alterations generally precede structural brain changes, diffusion parameters are expected to have higher predictive power than conventional morphometric measures.

The cerebellum appeared in many of our regression models, notably the quadrangular lobule of the cerebellum and lingula of the vermis. The basal ganglia are characterized by a relevant disynaptic projection to the cerebellar cortex [31]. Patients of PD with either tremor [32] or pain [33] showed damage in these regions. Reciprocal connections between the basal ganglia and cerebellum have been identified and may account for some of the clinical symptoms observed in PD [34]. Our study might suggest that the cerebellum could play an essential role in the disease progression of the motor symptoms in PD.

Regions found to be predictive of prognosis could form the keynodes within an interconnected network, for example, frontal-hippocampus and cerebellum-basal ganglia–thalamus networks. Brain connectivity within a whole neural network is altered in PD patients [35]. Therefore, affected regions might extend beyond observed pathogenesis, leading to changes in related motor and higher cognitive functions [36]. Our approach, which comprehensively assessed the damage throughout the whole brain, might potentially provide a more accurate prediction of the functional decline.

### 4.3. Prediction of Non-Motor Functions

Interestingly, the functions of these brain regions reflect well-known alterations in memory, language, and executive functions that occur in PD [37]. Our findings also suggest that both the thalamus and frontal lobe may be involved in the prediction of LEDD and the hippocampus in the prediction of UPDRS I. The functions of these memory- and cognitive-related areas involve regulation of sensory–motor, emotional and memory functions [38,39], and socio-emotional processing [40]. Although PD is predominantly considered to be a motor disorder, the extensive brain regions identified in our regression analysis supports the hypothesis that the disease process is multifocal in nature [41].

Because non-dopaminergic and non-motor symptoms tend to emerge later during the course of PD, their potential role as targets of cognitive training is attracting increasing attention [42]. Therefore, in order to effectively predict clinical outcome, it would be reasonable to assess the damage from a comprehensively neural network, rather than a few selected regions of interest.

Because non-motor symptoms in PD often precede motor abnormalities such as cognitive impairment, and are associated with worse prognosis [43], patients should be carefully monitored for potential early interventions [44,45]. We show that the clinical outcome in motor and non-motor symptoms can be confidently predicted by the diffusion characteristics in a combination of multiple brain regions. Our study might provide novel evidence of diffusion MRI as an effective and non-invasive prognostic tool.

### 4.4. Study Limitation

The main limitation in our study is the nature of our data-driven approach that enters the diffusion parameters into regression models, which makes it difficult to establish a direct causal relationship between the underlying function of an affected brain region and longitudinal changes in clinical severity. Further research aimed at disentangling the complex neural networks associated with clinical deterioration over time is therefore required.

Because only 82 patients were included in the final analysis, the generalizability of the study might be restricted. Our study presents a method, which selects features from the whole brain rather than a few pathophysiology related areas, to effectively predict the clinical outcome of patients with PD. Our observations of the involvement from additional brain regions might prompt further research into the role of progressive diffuse microstructural brain damage in the natural history of PD and to investigate the potential effect from chronic pharmacotherapy with an increased number of participants in the future study.

## 5. Conclusions

In conclusion, baseline parameters from diffusion tensor measurements from the whole brain of PD patients can accurately predict the decline in clinical outcomes over a 2-year period in individual patients, notably in the total and Category II of UPDRS.

## Figures and Tables

**Table 1:** General characteristics of participants and changes in clinical scores over time.

	Entire cohort (*n* = 82)	
Sex (Male/Female)	45/37	
Age, years	60.9 (7.3)	
Disease duration, years	6.4 (5.4)	
BMI	23.9 (3.2)	
Education	10.4 (4.3)	
	Baseline	Follow-up
LEDD	686.8 (349.2)	821.0 (395.2) ^†^
UPDRS	27.6 (16.4)	33.7 (19.2) ^†^
I	1.6 (1.9)	1.8 (1.7)
II	7.1 (4.9)	10.5 (6.6) ^†^
III	17.3 (10.7)	19.8 (11.7) ^†^
IV	1.6 (2.1)	1.6 (2.2)
MHY	1.9 (0.8)	2.2 (1.0) ^†^
1	31	23
1.5	9	6
2	9	16
2.5	18	10
3	15	19
4	0	6
5	0	2
ADL	0.9 (0.1)	0.8 (0.1) ^†^
PDQ39SI	22.7 (18.8)	33.9 (24.5) ^†^
SF36SI	100.5 (6.2)	95.9 (7.9) ^†^

Data are presented as counts or means (standard deviations or range in parentheses). † *p* < 0.005. The reported age and disease duration are those recorded at baseline. BMI: body mass index; LEDD: Levodopa equivalent daily dose; UPDRS: Unified Parkinson Disease Rating Scale; MHY: Modified Hoehn and Yahr staging; ADL: activity of daily living; PDQ39SI: Summary Index of 39-item PD Questionnaire; SF-36SI: Summary Index of the Taiwanese version of the Medical Outcomes Study Short Form-36.

**Table 2:** Diffusion parameters in parcellated cortical regions over time. Table 2 shows average values of fractional anisotropy and mean diffusivity in the different parcellated cortical regions at baseline and at follow-up. Data are reported as means ± standard deviations.

	FA						MD					
	90th Percentile		50th Percentile		10th Percentile		90th Percentile		50th Percentile		10th Percentile	
Brain Region	Baseline	Follow-Up	Baseline	Follow-Up	Baseline	Follow-Up	Baseline	Follow-Up	Baseline	Follow-Up	Baseline	Follow-Up
**Precentral L**	0.71 ± 0.05	0.70 ± 0.05	0.33 ± 0.04	0.30 ± 0.04 **	0.11 ± 0.01	0.10 ± 0.04	1.60 ± 0.14	1.71 ± 0.12 **	0.71 ± 0.04	0.74 ± 0.03 **	0.37 ± 0.06	0.36 ± 0.05
**Precentral R**	0.67 ± 0.05	0.67 ± 0.05	0.29 ± 0.04	0.26 ± 0.04 **	0.10 ± 0.01	0.10 ± 0.03	1.74 ± 0.15	1.83 ± 0.11 **	0.76 ± 0.04	0.80 ± 0.04 **	0.40 ± 0.05	0.38 ± 0.04 **
**SuperiorFrontal L**	0.64 ± 0.06	0.61 ± 0.06 **	0.30 ± 0.04	0.26 ± 0.04 **	0.11 ± 0.02	0.10 ± 0.03	1.58 ± 0.18	1.73 ± 0.12 **	0.73 ± 0.04	0.77 ± 0.05 **	0.41 ± 0.05	0.42 ± 0.04
**SuperiorFrontal R**	0.60 ± 0.05	0.59 ± 0.05	0.28 ± 0.04	0.26 ± 0.04 **	0.11 ± 0.02	0.10 ± 0.03	1.58 ± 0.19	1.71 ± 0.11 **	0.76 ± 0.04	0.78 ± 0.04 **	0.44 ± 0.05	0.43 ± 0.04
**SuperiorFrontal, Orbital L**	0.58 ± 0.15	0.55 ± 0.09	0.27 ± 0.11	0.23 ± 0.05	0.11 ± 0.04	0.10 ± 0.04	1.50 ± 0.29	1.67 ± 0.24 **	0.76 ± 0.11	0.83 ± 0.07 **	0.45 ± 0.08	0.43 ± 0.08
**SuperiorFrontal, Orbital R**	0.53 ± 0.16	0.51 ± 0.09	0.26 ± 0.09	0.22 ± 0.04	0.11 ± 0.04	0.10 ± 0.04	1.50 ± 0.23	1.66 ± 0.22 **	0.79 ± 0.08	0.86 ± 0.06 **	0.47 ± 0.12	0.47 ± 0.08
**MiddleFrontal L**	0.59 ± 0.05	0.57 ± 0.05	0.26 ± 0.04	0.23 ± 0.04 **	0.10 ± 0.01	0.10 ± 0.04	1.57 ± 0.16	1.74 ± 0.11 **	0.76 ± 0.04	0.80 ± 0.04 **	0.44 ± 0.04	0.43 ± 0.04
**MiddleFrontal R**	0.55 ± 0.04	0.54 ± 0.05	0.24 ± 0.03	0.22 ± 0.04	0.10 ± 0.01	0.10 ± 0.04	1.58 ± 0.16	1.73 ± 0.13 **	0.80 ± 0.04	0.83 ± 0.05 **	0.48 ± 0.04	0.47 ± 0.04
**MiddleFrontal, Orbital L**	0.58 ± 0.14	0.52 ± 0.06	0.27 ± 0.06	0.24 ± 0.04	0.11 ± 0.02	0.11 ± 0.03	1.44 ± 0.41	1.46 ± 0.20	0.79 ± 0.21	0.80 ± 0.05	0.44 ± 0.09	0.46 ± 0.05
**MiddleFrontal, Orbital R**	0.51 ± 0.15	0.45 ± 0.07	0.23 ± 0.05	0.21 ± 0.05	0.10 ± 0.02	0.10 ± 0.04	1.51 ± 0.43	1.56 ± 0.21	0.88 ± 0.30	0.87 ± 0.06	0.48 ± 0.13	0.52 ± 0.06
**InferiorFrontal, Opercular L**	0.59 ± 0.08	0.62 ± 0.07	0.25 ± 0.05	0.26 ± 0.05	0.10 ± 0.01	0.10 ± 0.04	1.83 ± 0.43	1.74 ± 0.16	0.88 ± 0.33	0.80 ± 0.06	0.45 ± 0.07	0.43 ± 0.06
**InferiorFrontal, Opercular R**	0.53 ± 0.06	0.55 ± 0.07	0.23 ± 0.04	0.24 ± 0.05	0.10 ± 0.01	0.10 ± 0.04	1.78 ± 0.39	1.72 ± 0.17	0.88 ± 0.13	0.85 ± 0.07	0.50 ± 0.05	0.48 ± 0.05
**InferiorFrontal, Triangular L**	0.59 ± 0.08	0.57 ± 0.05	0.25 ± 0.03	0.24 ± 0.05	0.10 ± 0.01	0.10 ± 0.04	1.72 ± 0.30	1.73 ± 0.14	0.80 ± 0.07	0.81 ± 0.05	0.43 ± 0.07	0.44 ± 0.04
**InferiorFrontal, Triangular R**	0.58 ± 0.10	0.55 ± 0.05	0.25 ± 0.04	0.24 ± 0.04	0.10 ± 0.01	0.10 ± 0.04	1.71 ± 0.28	1.74 ± 0.15	0.82 ± 0.10	0.83 ± 0.04	0.44 ± 0.09	0.46 ± 0.05
**InferiorFrontal, Orbital L**	0.57 ± 0.13	0.56 ± 0.08	0.27 ± 0.08	0.25 ± 0.04	0.12 ± 0.02	0.12 ± 0.04	1.57 ± 0.29	1.59 ± 0.15	0.79 ± 0.10	0.82 ± 0.04	0.45 ± 0.09	0.44 ± 0.08
**InferiorFrontal, Orbital R**	0.55 ± 0.14	0.56 ± 0.10	0.25 ± 0.08	0.24 ± 0.05	0.12 ± 0.03	0.11 ± 0.04	1.64 ± 0.29	1.68 ± 0.18	0.84 ± 0.10	0.85 ± 0.05	0.45 ± 0.12	0.43 ± 0.10
**RolandicOperculum L**	0.58 ± 0.08	0.61 ± 0.08	0.25 ± 0.04	0.28 ± 0.05 **	0.11 ± 0.02	0.12 ± 0.04	1.82 ± 0.39	1.61 ± 0.23 **	0.88 ± 0.26	0.77 ± 0.07	0.42 ± 0.06	0.40 ± 0.06
**RolandicOperculum R**	0.53 ± 0.07	0.55 ± 0.06	0.24 ± 0.03	0.26 ± 0.05	0.11 ± 0.01	0.12 ± 0.04	1.76 ± 0.41	1.57 ± 0.24 **	0.84 ± 0.10	0.79 ± 0.06 **	0.45 ± 0.05	0.44 ± 0.05
**SupplementaryMotorArea L**	0.61 ± 0.08	0.60 ± 0.07	0.27 ± 0.03	0.27 ± 0.04	0.11 ± 0.01	0.11 ± 0.03	1.72 ± 0.24	1.66 ± 0.16	0.80 ± 0.06	0.79 ± 0.05	0.43 ± 0.06	0.40 ± 0.07 **
**SupplementaryMotorArea R**	0.62 ± 0.07	0.62 ± 0.05	0.30 ± 0.03	0.29 ± 0.04	0.12 ± 0.01	0.12 ± 0.04	1.58 ± 0.23	1.57 ± 0.15	0.76 ± 0.04	0.77 ± 0.03	0.43 ± 0.06	0.41 ± 0.05 **
**Olfactory L**	0.49 ± 0.17	0.45 ± 0.09	0.27 ± 0.12	0.23 ± 0.05	0.13 ± 0.02	0.12 ± 0.04	1.37 ± 0.33	1.39 ± 0.26	0.79 ± 0.15	0.83 ± 0.07	0.52 ± 0.14	0.55 ± 0.07
**Olfactory R**	0.52 ± 0.17	0.50 ± 0.10	0.29 ± 0.09	0.27 ± 0.05	0.14 ± 0.03	0.14 ± 0.04	1.17 ± 0.29	1.18 ± 0.22	0.77 ± 0.12	0.79 ± 0.07	0.51 ± 0.13	0.53 ± 0.07
**SuperiorFrontal, Medial L**	0.50 ± 0.08	0.50 ± 0.05	0.21 ± 0.03	0.22 ± 0.04	0.09 ± 0.01	0.10 ± 0.04	1.87 ± 0.25	1.86 ± 0.14	0.85 ± 0.09	0.83 ± 0.04	0.46 ± 0.08	0.46 ± 0.04
**SuperiorFrontal, Medial R**	0.51 ± 0.07	0.51 ± 0.06	0.24 ± 0.02	0.23 ± 0.04	0.10 ± 0.01	0.10 ± 0.04	1.65 ± 0.20	1.78 ± 0.18 **	0.80 ± 0.04	0.82 ± 0.05	0.46 ± 0.07	0.46 ± 0.05
**SuperiorFrontal, MedialOrbital L**	0.48 ± 0.14	0.44 ± 0.07	0.22 ± 0.10	0.19 ± 0.05	0.10 ± 0.04	0.10 ± 0.04	1.75 ± 0.32	1.91 ± 0.26 **	0.85 ± 0.13	0.90 ± 0.08	0.50 ± 0.08	0.51 ± 0.06
**SuperiorFrontal, MedialOrbital R**	0.51 ± 0.14	0.47 ± 0.06	0.24 ± 0.08	0.22 ± 0.04	0.11 ± 0.03	0.10 ± 0.03	1.51 ± 0.26	1.63 ± 0.26 **	0.79 ± 0.10	0.83 ± 0.06	0.50 ± 0.07	0.51 ± 0.05
**Rectus L**	0.60 ± 0.15	0.54 ± 0.08	0.29 ± 0.09	0.24 ± 0.05	0.13 ± 0.03	0.11 ± 0.04	1.50 ± 0.26	1.65 ± 0.25 **	0.76 ± 0.10	0.83 ± 0.07 **	0.42 ± 0.09	0.43 ± 0.06
**Rectus R**	0.59 ± 0.14	0.54 ± 0.07	0.29 ± 0.08	0.25 ± 0.04	0.13 ± 0.03	0.12 ± 0.04	1.28 ± 0.26	1.45 ± 0.30 **	0.76 ± 0.09	0.80 ± 0.06 **	0.43 ± 0.10	0.44 ± 0.06
**Insula L**	0.49 ± 0.06	0.48 ± 0.05	0.24 ± 0.02	0.22 ± 0.04	0.11 ± 0.01	0.11 ± 0.04	1.69 ± 0.43	1.73 ± 0.21	0.84 ± 0.08	0.87 ± 0.06	0.48 ± 0.04	0.49 ± 0.04
**Insula R**	0.45 ± 0.07	0.43 ± 0.05	0.21 ± 0.03	0.20 ± 0.05	0.11 ± 0.01	0.10 ± 0.04	1.90 ± 0.34	1.97 ± 0.19	0.95 ± 0.13	1.04 ± 0.12 **	0.51 ± 0.05	0.52 ± 0.04
**Cingulum, Anterior L**	0.50 ± 0.10	0.43 ± 0.06 **	0.21 ± 0.02	0.20 ± 0.05	0.10 ± 0.01	0.10 ± 0.04	1.72 ± 0.30	1.76 ± 0.19	0.87 ± 0.09	0.88 ± 0.06	0.52 ± 0.08	0.54 ± 0.04
**Cingulum, Anterior R**	0.50 ± 0.07	0.47 ± 0.05	0.23 ± 0.02	0.23 ± 0.04	0.11 ± 0.01	0.11 ± 0.04	1.44 ± 0.33	1.39 ± 0.24	0.80 ± 0.05	0.80 ± 0.05	0.53 ± 0.05	0.53 ± 0.04
**Cingulum, Middle L**	0.68 ± 0.08	0.60 ± 0.07 **	0.27 ± 0.04	0.24 ± 0.05	0.12 ± 0.02	0.11 ± 0.04	1.62 ± 0.41	1.65 ± 0.19	0.91 ± 0.46	0.83 ± 0.05	0.46 ± 0.09	0.46 ± 0.04
**Cingulum, Middle R**	0.69 ± 0.08	0.60 ± 0.06 **	0.29 ± 0.05	0.26 ± 0.04	0.13 ± 0.02	0.12 ± 0.04	1.48 ± 0.46	1.53 ± 0.19	0.88 ± 0.45	0.79 ± 0.04	0.45 ± 0.09	0.45 ± 0.04
**Cingulum, Posterior L**	0.83 ± 0.08	0.77 ± 0.09 **	0.37 ± 0.08	0.33 ± 0.06	0.15 ± 0.02	0.14 ± 0.05	1.46 ± 0.38	1.38 ± 0.24	0.77 ± 0.17	0.75 ± 0.06	0.40 ± 0.07	0.43 ± 0.06
**Cingulum, Posterior R**	0.91 ± 0.07	0.90 ± 0.08	0.50 ± 0.13	0.47 ± 0.09	0.19 ± 0.04	0.18 ± 0.04	1.31 ± 0.46	1.19 ± 0.21	0.74 ± 0.28	0.66 ± 0.06	0.35 ± 0.07	0.35 ± 0.07
**Hippocampus L**	0.49 ± 0.09	0.47 ± 0.06	0.26 ± 0.03	0.26 ± 0.05	0.14 ± 0.01	0.14 ± 0.04	1.77 ± 0.26	1.84 ± 0.27	0.99 ± 0.14	1.00 ± 0.17	0.55 ± 0.10	0.56 ± 0.05
**Hippocampus R**	0.50 ± 0.10	0.48 ± 0.07	0.26 ± 0.03	0.25 ± 0.05	0.14 ± 0.02	0.14 ± 0.04	1.79 ± 0.24	1.88 ± 0.24 **	0.99 ± 0.17	1.02 ± 0.15	0.54 ± 0.11	0.56 ± 0.06
**ParaHippocampus L**	0.71 ± 0.10	0.68 ± 0.07	0.33 ± 0.03	0.34 ± 0.04	0.16 ± 0.01	0.17 ± 0.03	1.62 ± 0.31	1.60 ± 0.21	0.75 ± 0.09	0.74 ± 0.05	0.35 ± 0.08	0.37 ± 0.06
**ParaHippocampus R**	0.70 ± 0.11	0.68 ± 0.07	0.33 ± 0.05	0.34 ± 0.04	0.16 ± 0.02	0.17 ± 0.04	1.39 ± 0.29	1.30 ± 0.21	0.72 ± 0.09	0.71 ± 0.05	0.36 ± 0.08	0.36 ± 0.06
**Amygdala L**	0.51 ± 0.08	0.53 ± 0.09	0.29 ± 0.04	0.30 ± 0.05	0.16 ± 0.02	0.16 ± 0.04	1.66 ± 0.30	1.67 ± 0.29	0.80 ± 0.12	0.78 ± 0.07	0.48 ± 0.08	0.47 ± 0.08
**Amygdala R**	0.54 ± 0.08	0.54 ± 0.08	0.31 ± 0.04	0.31 ± 0.05	0.17 ± 0.02	0.17 ± 0.04	1.47 ± 0.24	1.49 ± 0.31	0.77 ± 0.05	0.76 ± 0.06	0.47 ± 0.07	0.47 ± 0.08
**Calcarine L**	0.49 ± 0.07	0.50 ± 0.06	0.19 ± 0.02	0.20 ± 0.05	0.09 ± 0.01	0.09 ± 0.04	1.75 ± 0.17	1.80 ± 0.17	0.92 ± 0.08	0.94 ± 0.09	0.50 ± 0.06	0.51 ± 0.05
**Calcarine R**	0.57 ± 0.07	0.57 ± 0.06	0.24 ± 0.03	0.25 ± 0.04	0.10 ± 0.01	0.10 ± 0.04	1.60 ± 0.19	1.64 ± 0.20	0.83 ± 0.06	0.84 ± 0.06	0.48 ± 0.06	0.49 ± 0.04
**Cuneus L**	0.49 ± 0.05	0.50 ± 0.04	0.18 ± 0.02	0.19 ± 0.04	0.08 ± 0.01	0.08 ± 0.03	1.76 ± 0.17	1.78 ± 0.16	0.91 ± 0.08	0.91 ± 0.07	0.50 ± 0.04	0.51 ± 0.04
**Cuneus R**	0.57 ± 0.05	0.57 ± 0.05	0.24 ± 0.03	0.23 ± 0.05	0.09 ± 0.01	0.09 ± 0.04	1.60 ± 0.20	1.65 ± 0.20	0.80 ± 0.06	0.81 ± 0.06	0.47 ± 0.05	0.47 ± 0.04
**Lingual L**	0.54 ± 0.06	0.55 ± 0.05	0.26 ± 0.03	0.26 ± 0.04	0.12 ± 0.01	0.12 ± 0.04	1.45 ± 0.23	1.51 ± 0.20	0.80 ± 0.09	0.80 ± 0.05	0.48 ± 0.05	0.46 ± 0.04
**Lingual R**	0.61 ± 0.08	0.63 ± 0.06	0.30 ± 0.04	0.29 ± 0.04	0.13 ± 0.02	0.13 ± 0.04	1.39 ± 0.28	1.48 ± 0.22	0.77 ± 0.12	0.77 ± 0.05	0.42 ± 0.07	0.39 ± 0.06
**SuperiorOccipital L**	0.58 ± 0.06	0.56 ± 0.05	0.26 ± 0.05	0.22 ± 0.05 **	0.09 ± 0.01	0.08 ± 0.04	1.54 ± 0.20	1.70 ± 0.21 **	0.78 ± 0.05	0.83 ± 0.05 **	0.48 ± 0.06	0.48 ± 0.05
**SuperiorOccipital R**	0.62 ± 0.06	0.60 ± 0.06	0.26 ± 0.05	0.22 ± 0.05 **	0.09 ± 0.01	0.08 ± 0.04	1.54 ± 0.21	1.70 ± 0.20 **	0.77 ± 0.04	0.83 ± 0.06 **	0.47 ± 0.05	0.46 ± 0.05
**MiddleOccipital L**	0.53 ± 0.05	0.47 ± 0.04 **	0.23 ± 0.04	0.20 ± 0.04 **	0.09 ± 0.01	0.08 ± 0.04	1.38 ± 0.19	1.54 ± 0.19 **	0.78 ± 0.04	0.83 ± 0.05 **	0.53 ± 0.04	0.55 ± 0.03 **
**MiddleOccipital R**	0.51 ± 0.06	0.48 ± 0.05 **	0.22 ± 0.03	0.19 ± 0.04 **	0.09 ± 0.01	0.09 ± 0.04	1.43 ± 0.20	1.58 ± 0.20 **	0.80 ± 0.05	0.86 ± 0.06 **	0.51 ± 0.05	0.52 ± 0.04
**InferiorOccipital L**	0.50 ± 0.06	0.47 ± 0.05 **	0.26 ± 0.04	0.23 ± 0.04 **	0.11 ± 0.02	0.10 ± 0.04	1.20 ± 0.29	1.37 ± 0.20 **	0.78 ± 0.13	0.80 ± 0.04	0.54 ± 0.07	0.55 ± 0.04
**InferiorOccipital R**	0.51 ± 0.09	0.50 ± 0.09	0.24 ± 0.04	0.23 ± 0.05	0.11 ± 0.01	0.11 ± 0.04	1.31 ± 0.26	1.45 ± 0.20 **	0.78 ± 0.09	0.83 ± 0.08	0.50 ± 0.09	0.49 ± 0.07
**Fusiform L**	0.57 ± 0.06	0.55 ± 0.05	0.29 ± 0.03	0.28 ± 0.04	0.14 ± 0.02	0.14 ± 0.03	1.24 ± 0.29	1.32 ± 0.18	0.76 ± 0.05	0.77 ± 0.04	0.45 ± 0.07	0.46 ± 0.05
**Fusiform R**	0.65 ± 0.08	0.63 ± 0.06	0.32 ± 0.04	0.31 ± 0.04	0.15 ± 0.02	0.15 ± 0.04	1.17 ± 0.31	1.25 ± 0.20	0.71 ± 0.06	0.73 ± 0.05	0.37 ± 0.09	0.39 ± 0.05
**Postcentral L**	0.67 ± 0.05	0.65 ± 0.05 **	0.28 ± 0.04	0.25 ± 0.04 **	0.10 ± 0.01	0.10 ± 0.04	1.76 ± 0.17	1.81 ± 0.13	0.79 ± 0.04	0.83 ± 0.04 **	0.39 ± 0.05	0.38 ± 0.05
**Postcentral R**	0.64 ± 0.05	0.61 ± 0.05 **	0.25 ± 0.04	0.22 ± 0.05 **	0.09 ± 0.01	0.09 ± 0.04	1.77 ± 0.15	1.83 ± 0.13	0.82 ± 0.05	0.88 ± 0.06 **	0.42 ± 0.05	0.41 ± 0.05
**SuperiorParietal L**	0.59 ± 0.06	0.53 ± 0.05 **	0.25 ± 0.05	0.20 ± 0.04 **	0.08 ± 0.02	0.08 ± 0.04	1.69 ± 0.19	1.83 ± 0.13 **	0.81 ± 0.04	0.88 ± 0.05 **	0.47 ± 0.05	0.47 ± 0.05
**SuperiorParietal R**	0.57 ± 0.07	0.52 ± 0.07 **	0.22 ± 0.06	0.19 ± 0.05	0.08 ± 0.01	0.08 ± 0.04	1.81 ± 0.17	1.82 ± 0.12	0.86 ± 0.08	0.90 ± 0.06 **	0.46 ± 0.05	0.46 ± 0.07
**InferiorParietal L**	0.56 ± 0.05	0.53 ± 0.05 **	0.24 ± 0.04	0.20 ± 0.04 **	0.09 ± 0.01	0.08 ± 0.04	1.64 ± 0.19	1.77 ± 0.15 **	0.81 ± 0.05	0.87 ± 0.04 **	0.48 ± 0.04	0.47 ± 0.05
**InferiorParietal R**	0.54 ± 0.07	0.52 ± 0.06	0.20 ± 0.05	0.18 ± 0.05	0.08 ± 0.01	0.08 ± 0.04	1.68 ± 0.20	1.79 ± 0.17 **	0.85 ± 0.08	0.91 ± 0.09 **	0.48 ± 0.06	0.48 ± 0.06
**SupraMarginal L**	0.54 ± 0.05	0.52 ± 0.06	0.24 ± 0.03	0.23 ± 0.05	0.10 ± 0.01	0.10 ± 0.04	1.59 ± 0.31	1.66 ± 0.20	0.82 ± 0.08	0.84 ± 0.06	0.47 ± 0.05	0.48 ± 0.05
**SupraMarginal R**	0.51 ± 0.06	0.50 ± 0.05	0.20 ± 0.02	0.19 ± 0.04	0.09 ± 0.01	0.08 ± 0.04	1.69 ± 0.24	1.74 ± 0.14	0.84 ± 0.05	0.87 ± 0.05 **	0.48 ± 0.06	0.49 ± 0.05
**Angular L**	0.52 ± 0.05	0.48 ± 0.05 **	0.23 ± 0.04	0.20 ± 0.04 **	0.08 ± 0.01	0.08 ± 0.04	1.45 ± 0.20	1.62 ± 0.18 **	0.81 ± 0.05	0.85 ± 0.06 **	0.56 ± 0.04	0.56 ± 0.04
**Angular R**	0.52 ± 0.06	0.50 ± 0.06	0.21 ± 0.03	0.19 ± 0.04	0.09 ± 0.01	0.08 ± 0.04	1.51 ± 0.19	1.64 ± 0.15 **	0.82 ± 0.06	0.87 ± 0.05 **	0.53 ± 0.05	0.53 ± 0.06
**Precuneus L**	0.50 ± 0.06	0.52 ± 0.05	0.20 ± 0.02	0.20 ± 0.04	0.09 ± 0.01	0.09 ± 0.04	1.74 ± 0.17	1.71 ± 0.16	0.87 ± 0.04	0.86 ± 0.04	0.50 ± 0.05	0.49 ± 0.05
**Precuneus R**	0.57 ± 0.07	0.57 ± 0.05	0.23 ± 0.03	0.23 ± 0.04	0.09 ± 0.01	0.09 ± 0.04	1.65 ± 0.18	1.69 ± 0.15	0.82 ± 0.05	0.83 ± 0.04	0.46 ± 0.05	0.46 ± 0.04
**Paracentralobule L**	0.65 ± 0.07	0.65 ± 0.07	0.31 ± 0.03	0.29 ± 0.04	0.11 ± 0.01	0.10 ± 0.03	1.63 ± 0.21	1.70 ± 0.19	0.74 ± 0.04	0.76 ± 0.04	0.40 ± 0.07	0.37 ± 0.07
**Paracentralobule R**	0.60 ± 0.08	0.59 ± 0.08	0.26 ± 0.04	0.25 ± 0.05	0.10 ± 0.01	0.10 ± 0.04	1.70 ± 0.25	1.74 ± 0.21	0.79 ± 0.08	0.81 ± 0.08	0.39 ± 0.08	0.37 ± 0.08
**Caudate L**	0.66 ± 0.10	0.58 ± 0.10 **	0.29 ± 0.05	0.26 ± 0.06 **	0.13 ± 0.02	0.12 ± 0.05	1.90 ± 0.42	2.17 ± 0.37 **	0.81 ± 0.14	0.96 ± 0.25 **	0.36 ± 0.08	0.42 ± 0.11 **
**Caudate R**	0.62 ± 0.10	0.59 ± 0.10	0.30 ± 0.04	0.28 ± 0.06	0.14 ± 0.02	0.14 ± 0.05	1.66 ± 0.44	1.82 ± 0.49	0.75 ± 0.08	0.84 ± 0.22 **	0.37 ± 0.09	0.41 ± 0.10
**Putamen L**	0.83 ± 0.09	0.82 ± 0.09	0.46 ± 0.08	0.48 ± 0.06	0.24 ± 0.05	0.25 ± 0.04	0.96 ± 0.40	0.86 ± 0.10	0.58 ± 0.09	0.57 ± 0.08	0.22 ± 0.09	0.24 ± 0.09
**Putamen R**	0.76 ± 0.08	0.75 ± 0.07	0.43 ± 0.06	0.44 ± 0.05	0.23 ± 0.04	0.24 ± 0.03	1.00 ± 0.36	0.89 ± 0.08	0.64 ± 0.08	0.63 ± 0.06	0.29 ± 0.09	0.31 ± 0.08
**Pallidum L**	0.94 ± 0.15	0.98 ± 0.06	0.65 ± 0.16	0.68 ± 0.08	0.33 ± 0.09	0.36 ± 0.06	0.95 ± 0.56	0.79 ± 0.12	0.47 ± 0.29	0.40 ± 0.12	0.13 ± 0.17	0.08 ± 0.09
**Pallidum R**	0.92 ± 0.15	0.96 ± 0.07	0.60 ± 0.15	0.63 ± 0.09	0.31 ± 0.08	0.32 ± 0.06	0.99 ± 0.48	0.84 ± 0.13	0.50 ± 0.21	0.45 ± 0.11	0.13 ± 0.14	0.09 ± 0.07
**Thalamus L**	0.83 ± 0.06	0.80 ± 0.05	0.48 ± 0.05	0.46 ± 0.05	0.24 ± 0.04	0.22 ± 0.04	1.14 ± 0.51	1.27 ± 0.36	0.59 ± 0.08	0.61 ± 0.05	0.24 ± 0.06	0.27 ± 0.05
**Thalamus R**	0.82 ± 0.05	0.81 ± 0.05	0.48 ± 0.05	0.47 ± 0.05	0.25 ± 0.04	0.23 ± 0.04	1.07 ± 0.48	1.14 ± 0.33	0.60 ± 0.10	0.60 ± 0.05	0.25 ± 0.05	0.25 ± 0.05
**Heschl L**	0.63 ± 0.12	0.61 ± 0.13	0.28 ± 0.08	0.26 ± 0.08	0.13 ± 0.03	0.12 ± 0.05	1.81 ± 0.33	1.93 ± 0.28	0.92 ± 0.22	1.00 ± 0.29	0.38 ± 0.12	0.37 ± 0.12
**Heschl R**	0.64 ± 0.14	0.66 ± 0.12	0.28 ± 0.09	0.28 ± 0.08	0.13 ± 0.04	0.12 ± 0.05	1.76 ± 0.40	1.87 ± 0.38	0.89 ± 0.25	0.87 ± 0.27	0.33 ± 0.11	0.32 ± 0.12
**SuperiorTemporal L**	0.64 ± 0.06	0.62 ± 0.05	0.27 ± 0.03	0.25 ± 0.05	0.11 ± 0.01	0.11 ± 0.04	1.67 ± 0.19	1.77 ± 0.16 **	0.78 ± 0.06	0.79 ± 0.05	0.37 ± 0.05	0.37 ± 0.04
**SuperiorTemporal R**	0.62 ± 0.06	0.61 ± 0.05	0.26 ± 0.02	0.26 ± 0.04	0.11 ± 0.01	0.11 ± 0.04	1.55 ± 0.15	1.63 ± 0.15 **	0.75 ± 0.04	0.77 ± 0.04 **	0.40 ± 0.05	0.41 ± 0.04
**SuperiorTemporalPole L**	0.46 ± 0.11	0.44 ± 0.06	0.21 ± 0.04	0.21 ± 0.04	0.10 ± 0.02	0.10 ± 0.04	1.99 ± 0.27	2.03 ± 0.19	0.93 ± 0.15	0.91 ± 0.08	0.48 ± 0.09	0.50 ± 0.06
**SuperiorTemporalPole R**	0.46 ± 0.11	0.46 ± 0.06	0.23 ± 0.06	0.23 ± 0.04	0.11 ± 0.01	0.11 ± 0.04	1.75 ± 0.23	1.77 ± 0.18	0.84 ± 0.09	0.84 ± 0.06	0.49 ± 0.06	0.49 ± 0.06
**MiddleTemporal L**	0.57 ± 0.06	0.54 ± 0.04 **	0.25 ± 0.02	0.23 ± 0.04	0.11 ± 0.01	0.10 ± 0.04	1.40 ± 0.18	1.52 ± 0.16 **	0.77 ± 0.04	0.80 ± 0.04 **	0.44 ± 0.06	0.46 ± 0.04
**MiddleTemporal R**	0.52 ± 0.08	0.50 ± 0.06	0.22 ± 0.02	0.22 ± 0.04	0.10 ± 0.01	0.10 ± 0.04	1.42 ± 0.17	1.52 ± 0.16 **	0.79 ± 0.05	0.82 ± 0.05 **	0.46 ± 0.09	0.48 ± 0.05
**MiddleTemporalPole L**	0.52 ± 0.14	0.47 ± 0.06	0.27 ± 0.10	0.25 ± 0.04	0.13 ± 0.05	0.12 ± 0.04	1.40 ± 0.29	1.44 ± 0.24	0.78 ± 0.13	0.81 ± 0.06	0.49 ± 0.09	0.51 ± 0.06
**MiddleTemporalPole R**	0.56 ± 0.13	0.49 ± 0.08	0.29 ± 0.10	0.25 ± 0.04	0.13 ± 0.03	0.12 ± 0.03	1.28 ± 0.21	1.36 ± 0.21	0.75 ± 0.11	0.80 ± 0.05	0.47 ± 0.10	0.50 ± 0.07
**InferiorTemporal L**	0.68 ± 0.11	0.61 ± 0.05 **	0.31 ± 0.04	0.28 ± 0.04 **	0.13 ± 0.01	0.13 ± 0.04	1.17 ± 0.25	1.26 ± 0.18 **	0.71 ± 0.06	0.75 ± 0.04 **	0.39 ± 0.08	0.42 ± 0.04
**InferiorTemporal R**	0.62 ± 0.13	0.57 ± 0.06	0.28 ± 0.06	0.26 ± 0.04	0.12 ± 0.01	0.12 ± 0.04	1.27 ± 0.28	1.37 ± 0.17	0.73 ± 0.09	0.78 ± 0.04 **	0.43 ± 0.06	0.44 ± 0.04
**SupeiorSemilunar L**	0.36 ± 0.06	0.37 ± 0.07	0.19 ± 0.02	0.20 ± 0.04	0.10 ± 0.01	0.11 ± 0.04	1.80 ± 0.22	1.73 ± 0.18	0.97 ± 0.09	0.90 ± 0.11 **	0.57 ± 0.07	0.57 ± 0.05
**SupeiorSemilunar R**	0.45 ± 0.11	0.41 ± 0.09	0.21 ± 0.02	0.22 ± 0.04	0.11 ± 0.01	0.12 ± 0.03	1.78 ± 0.25	1.67 ± 0.21	0.92 ± 0.09	0.88 ± 0.13	0.49 ± 0.10	0.54 ± 0.12
**InfeiorSemilunar L**	0.39 ± 0.12	0.37 ± 0.10	0.21 ± 0.03	0.21 ± 0.06	0.11 ± 0.02	0.12 ± 0.04	1.63 ± 0.24	1.58 ± 0.30	0.84 ± 0.07	0.91 ± 0.24	0.54 ± 0.10	0.60 ± 0.17
**InfeiorSemilunar R**	0.42 ± 0.11	0.37 ± 0.12	0.22 ± 0.04	0.21 ± 0.05	0.12 ± 0.02	0.12 ± 0.04	1.67 ± 0.26	1.63 ± 0.37	0.86 ± 0.23	1.00 ± 0.39	0.50 ± 0.11	0.64 ± 0.38
**Alae L**	0.51 ± 0.18	0.47 ± 0.13	0.25 ± 0.10	0.23 ± 0.06	0.14 ± 0.05	0.13 ± 0.05	2.17 ± 0.43	2.14 ± 0.24	1.19 ± 0.35	1.31 ± 0.31	0.51 ± 0.18	0.60 ± 0.22
**Alae R**	0.55 ± 0.18	0.56 ± 0.14	0.28 ± 0.10	0.27 ± 0.06	0.15 ± 0.06	0.14 ± 0.04	2.02 ± 0.42	2.02 ± 0.28	1.02 ± 0.32	1.08 ± 0.29	0.43 ± 0.16	0.43 ± 0.14
**Quadrangular L**	0.50 ± 0.14	0.50 ± 0.07	0.27 ± 0.09	0.26 ± 0.04	0.13 ± 0.02	0.14 ± 0.03	1.68 ± 0.25	1.58 ± 0.24	0.85 ± 0.12	0.80 ± 0.08	0.47 ± 0.09	0.45 ± 0.06
**Quadrangular R**	0.51 ± 0.16	0.51 ± 0.08	0.28 ± 0.10	0.27 ± 0.05	0.14 ± 0.02	0.15 ± 0.04	1.79 ± 0.25	1.64 ± 0.25 **	0.89 ± 0.15	0.83 ± 0.09	0.47 ± 0.10	0.45 ± 0.07
**LobulusSimplex L**	0.39 ± 0.09	0.38 ± 0.05	0.22 ± 0.04	0.22 ± 0.04	0.12 ± 0.02	0.12 ± 0.03	1.60 ± 0.22	1.53 ± 0.20	0.89 ± 0.10	0.83 ± 0.07	0.55 ± 0.07	0.54 ± 0.04
**LobulusSimplex R**	0.44 ± 0.11	0.43 ± 0.05	0.25 ± 0.05	0.25 ± 0.04	0.13 ± 0.02	0.14 ± 0.03	1.53 ± 0.20	1.39 ± 0.19 **	0.84 ± 0.10	0.78 ± 0.06 **	0.51 ± 0.08	0.50 ± 0.05
**Biventral L**	0.51 ± 0.11	0.39 ± 0.12 **	0.29 ± 0.07	0.23 ± 0.07 **	0.16 ± 0.04	0.14 ± 0.05	1.08 ± 0.48	1.48 ± 0.51 **	0.70 ± 0.11	0.96 ± 0.38 **	0.49 ± 0.10	0.65 ± 0.33
**Biventral R**	0.54 ± 0.13	0.41 ± 0.15 **	0.29 ± 0.06	0.24 ± 0.07 **	0.16 ± 0.04	0.13 ± 0.05	1.13 ± 0.47	1.47 ± 0.47 **	0.73 ± 0.19	0.95 ± 0.37 **	0.47 ± 0.12	0.63 ± 0.32
**Biventral L**	0.66 ± 0.11	0.52 ± 0.13 **	0.38 ± 0.06	0.28 ± 0.07 **	0.18 ± 0.04	0.15 ± 0.04	1.08 ± 0.38	1.32 ± 0.43	0.64 ± 0.07	0.81 ± 0.29 **	0.40 ± 0.08	0.51 ± 0.21
**Biventral R**	0.72 ± 0.12	0.62 ± 0.16	0.41 ± 0.07	0.34 ± 0.09 **	0.20 ± 0.03	0.18 ± 0.06	0.92 ± 0.27	1.12 ± 0.34	0.61 ± 0.09	0.71 ± 0.24	0.34 ± 0.08	0.43 ± 0.23
**Tonsil L**	0.76 ± 0.11	0.64 ± 0.13 **	0.39 ± 0.07	0.31 ± 0.07 **	0.17 ± 0.03	0.16 ± 0.04	1.52 ± 0.33	1.74 ± 0.30 **	0.69 ± 0.15	0.83 ± 0.23 **	0.32 ± 0.09	0.40 ± 0.17
**Tonsil R**	0.76 ± 0.12	0.66 ± 0.14 **	0.40 ± 0.06	0.33 ± 0.07 **	0.18 ± 0.03	0.16 ± 0.04	1.41 ± 0.24	1.64 ± 0.31 **	0.66 ± 0.10	0.80 ± 0.22 **	0.31 ± 0.12	0.36 ± 0.16
**Flocculus L**	0.86 ± 0.19	0.60 ± 0.20 **	0.58 ± 0.17	0.29 ± 0.09 **	0.23 ± 0.10	0.14 ± 0.05 **	1.54 ± 0.50	2.00 ± 0.39 **	0.69 ± 0.27	1.08 ± 0.35 **	0.35 ± 0.22	0.47 ± 0.28
**Flocculus R**	0.84 ± 0.16	0.57 ± 0.20 **	0.53 ± 0.18	0.25 ± 0.10 **	0.20 ± 0.12	0.13 ± 0.05 **	1.67 ± 0.51	2.02 ± 0.43 **	0.75 ± 0.28	1.15 ± 0.30 **	0.40 ± 0.23	0.51 ± 0.21
**Lingula**	0.44 ± 0.17	0.47 ± 0.15	0.23 ± 0.08	0.26 ± 0.08	0.13 ± 0.06	0.15 ± 0.07	1.92 ± 0.46	1.90 ± 0.32	1.25 ± 0.40	1.25 ± 0.36	0.75 ± 0.35	0.75 ± 0.31
**CentralLobule**	0.49 ± 0.13	0.50 ± 0.11	0.23 ± 0.04	0.24 ± 0.05	0.12 ± 0.02	0.13 ± 0.04	2.16 ± 0.26	2.07 ± 0.15	1.22 ± 0.38	1.11 ± 0.19	0.50 ± 0.12	0.49 ± 0.12
**Culmen**	0.43 ± 0.11	0.44 ± 0.07	0.22 ± 0.05	0.22 ± 0.05	0.12 ± 0.02	0.12 ± 0.04	1.85 ± 0.27	1.79 ± 0.17	0.99 ± 0.14	0.95 ± 0.09	0.55 ± 0.10	0.54 ± 0.07
**Declive**	0.38 ± 0.09	0.35 ± 0.07	0.18 ± 0.03	0.18 ± 0.05	0.10 ± 0.02	0.10 ± 0.05	2.05 ± 0.28	1.89 ± 0.16 **	1.20 ± 0.20	1.11 ± 0.13	0.62 ± 0.11	0.63 ± 0.08
**FoliumTuber**	0.33 ± 0.07	0.41 ± 0.07 **	0.19 ± 0.03	0.23 ± 0.05 **	0.10 ± 0.02	0.12 ± 0.04	1.75 ± 0.29	1.55 ± 0.29 **	1.07 ± 0.21	0.86 ± 0.11 **	0.67 ± 0.13	0.57 ± 0.09 **
**Pyramid**	0.49 ± 0.09	0.54 ± 0.10	0.27 ± 0.04	0.30 ± 0.06 **	0.14 ± 0.02	0.16 ± 0.04	1.22 ± 0.41	1.10 ± 0.20	0.78 ± 0.24	0.69 ± 0.06	0.48 ± 0.08	0.45 ± 0.07
**Uvula**	0.68 ± 0.12	0.61 ± 0.13	0.39 ± 0.08	0.33 ± 0.07 **	0.21 ± 0.05	0.17 ± 0.06 **	1.01 ± 0.45	1.37 ± 0.32 **	0.65 ± 0.25	0.71 ± 0.11	0.36 ± 0.11	0.37 ± 0.11
**Nodule**	0.66 ± 0.16	0.53 ± 0.19 **	0.35 ± 0.10	0.24 ± 0.06 **	0.18 ± 0.05	0.13 ± 0.03 **	1.71 ± 0.51	2.19 ± 0.31 **	0.79 ± 0.26	1.30 ± 0.41 **	0.37 ± 0.16	0.62 ± 0.33 **

Diffusivity is expressed in 10^−3^ mm^2^/sec; **, denotes *p* < 0.01 with correction for multiple comparison by using Bonferroni method (0.01/696); L, left hemisphere; R, right hemisphere; MD, mean diffusivity, FA, fractional anisotropy.

**Table 3:** Statistical results of each assessment from the regression model. The statistical results, including adjusted R2 and F values, are depicted for each assessment. The averaged adjusted R2 and the mean average errors between predicted and observed values are calculated from cross validation and blind validation.

	UPDRS_TOTAL	LEDD	PDQ39 SI	MHY	UPDRSI	UPDRSII	UPDRSIII	UPDRSIV
**Adjusted R^2^**	0.94	0.89	0.88	0.86	0.57	0.92	0.93	0.92
**F Test**	994	528	479	390	87.9	780	834	716
**LOOCV**								
**Mean Adjusted R^2^**	0.939 ± 0.002	0.891 ± 0.004	0.881 ± 0.004	0.871 ± 0.003	0.573 ± 0.012	0.923 ± 0.003	0.928 ± 0.002	0.917 ± 0.006
**MAE**	4.39 ± 3.57	138.70 ± 112.38	8.08 ± 5.39	0.40 ± 0.25	0.97 ± 0.76	1.74 ± 1.31	3.21 ± 2.31	0.61 ± 0.48
**5-fold CV**								
**Mean Adjusted R^2^**	0.944 ± 0.011	0.898 ± 0.006	0.882 ± 0.013	0.877 ± 0.009	0.592 ± 0.051	0.930 ± 0.007	0.933 ± 0.005	0.919 ± 0.021
**MAE**	4.94 ± 1.67	135.64 ± 23.99	7.15 ± 1.32	0.39 ± 0.07	1.06 ± 0.15	2.05 ± 0.46	3.35 ± 0.33	0.67 ± 0.12
**Blind validation**								
**Adjusted R^2^**	0.76	0.42	0.54	0.1	0.01	0.82	0.62	0.32
**MAE**	8.72 ± 6.24	348.17 ± 202.22	17.44 ± 11.44	1.02 ± 0.93	2.19 ± 2.28	3.06 ± 2.24	7.46 ± 4.07	2.10 ± 1.77
**MAE in %**	4.38 ± 3.14	53.09 ± 44.45	11.18 ± 7.33	20.40 ± 18.63	13.66 ± 14.27	5.88 ± 4.31	6.90 ± 3.77	9.11 ± 7.68

LOOCV, leave-one-out cross validation; MAE: mean absolute error; UPDRS: Unified Parkinson’s Disease Rating Scale; MHY: Modified Hoehn and Yahr staging; LEDD: levodopa equivalent daily dose; PDQ39SI: Summary Index of 39-item PD Questionnaire. The percentage change in MAE (MAE in %) was calculated as MAE divided by the maximum range from the corresponding assessment. However, in LEDD, MAE in % was calculated as MAE divided by the observed LEDD from each individual.

**Table 4:** Equation with dependent variables and the corresponding unstandardized coefficients in the regression model of UPDRS and its categories. Table 4 shows the dependent variables and corresponding unstandardized coefficients for the total score and Category I–IV of UPDRS from which clinical changes in individual patients can be calculated by the combination of diffusion parameters.

**UPDRS_TOTAL **=		
+67.84 * Nodule(FA_50_)	+48.82 * Tonsil *_L_*(MD_10_)	+11.53 * Lingula(MD_10_)
+79.50 * RolandicOperculum *_R_*(FA_50_)	+19.28 * Rectus *_L_*(MD_90_)	+222.21 * Quadrangular *_R_*(FA_10_)
−23.12 * Amygdala *_L_*(FA_90_)	+10.08 * Heschl *_L_*(MD_90_)	−7.38 * Flocculus *_R_*(MD_90_)
−80.25 * ParaHippocampus *_L_*(FA_50_)	+57.31 * Biventral *_R_*(FA_10_)	+60.29 * Cuneus *_R_*(FA_50_)
+6.09 * Alae *_R_*(MD_50_)	−118.61	
**UPDRS_I** =		
+3.64 * Hippocampus *_L_*(MD_90_)	+18.70 * Olfactory *_R_*(FA_10_)	+36.22 * SuperiorSemilunar *_R_*(FA_10_)
−1.83 * SuperiorFrontal, Medial *_R_*(MD_90_)	+12.01 * Thalamus *_R_*(MD_10_)	−31.55 * RolandicOperculum *_L_*(FA_10_)
+3.89 * Biventral *_R_*(FA_90_)	+19.83 * FoliumTuber(FA_10_)	−8.39 * SuperiorTemporalPole *_R_*(FA_50_)
−2.72 * SuperiorFrontal, Orbital *_L_*(MD_90_)	−44.07 * InferiorParietal *_R_*(FA_10_)	−6.69 * Culmen(MD_10_)
−3.82 * InferiorFrontal, Orbital *_R_*(FA_90_)	+1.74	
**UPDRS_II** =		
+8.87 * Nodule(FA_50_)	−9.99 * Lingual *_L_*(MD_10_)	−21.90 * Thalamus *_R_*(FA_50_)
+63.89 * RolandicOperculum *_R_*(FA_50_)	+60.38 * Quadrangular *_R_*(FA_10_)	−54.70 * Insula *_L_*(FA_50_)
+78.06 * Cingulum, Middle *_R_*(FA_10_)	+21.55 * Bivnetral *_R_*(FA_10_)	+4.78 * Hippocampus *_L_*(MD_90_)
−19.08 * Uvula(FA_10_)	+3.03 * Rectus *_L_*(MD_90_)	−4.79 * Heschl *_L_*(FA_90_)
+1.20 * Lingula(MD_10_)	−15.43	
**UPDRS_III** =		
+44.92 * Nodule(FA_50_)	+33.65 * Tonsil *_L_*(MD_10_)	−17.12 * Heschl *_R_*(FA_90_)
+2.39 * Lingula(MD_50_)	−6.27 * Flocculus *_R_*(MD_90_)	+18.90 * SuperiorTemporalPole *_R_*(MD_50_)
+93.69 * Quadrangular *_R_*(FA_10_)	−55.96 * ParaHippocampus *_L_*(FA_50_)	+8.14 * Heschl *_L_*(MD_90_)
−23.69 * Caudate *_R_*(MD_10_)	+11.13 * Rectus *_L_*(MD_90_)	+58.03 * Cuneus *_R_*(FA_50_)
+5.47 * Lingula(MD_10_)	−56.224	
**UPDRS_IV** =		
−0.87 * FoliumTuber(MD_90_)	−4.84 * CentralLobule (MD_10_)	−11.18 * Postcentral *_R_*(FA_90_)
+9.65 * InferiorOccipital *_R_*(FA_50_)	+28.43 * InferiorFrontal, Orbital *_L_*(FA_10_)	−23.17 * Caudate *_L_*(FA_10_)
+21.73 * RolandicOperculum *_R_*(FA_10_)	−31.30 * MiddleFrontal, Orbital *_R_*(FA_10_)	−1.34 * SuperiorFrontal, Orbital *_L_*(MD_90_)
−23.42 * LobulusSimplex *_L_*(FA_10_)	+11.19 * Cuneus *_L_*(FA_50_)	+16.83 * Cingulum, Anterior *_R_*(FA_50_)
−21.21 * InferiorFrontal, Opercular *_R_*(FA_10_)	+10.32	

UPDRS: Unified Parkinson’s Disease Rating Scale; MD, mean diffusivity; FA, fractional anisotropy; *R*, right hemisphere; *L*, left hemisphere; 90, 90th percentile; 50, 50th percentile; 10, 10th percentile; * indicated multiplication.

**Table 5:** Equation with dependent variables and the corresponding unstandardized coefficients in the regression model of LEDD, MHY, and PDQ39SI. Table 5 shows the dependent variables and corresponding unstandardized coefficients for LEDD, MHY, and PDQ39SI from which clinical changes in individual patients can be calculated by the combination of diffusion parameters.

**LEDD** =		
+1347.19 * ParacentralLobule *_R_*(MD_10_)	+3997.05 * InferiorFrontal, Opercular *_L_*(FA_10_)	+728.10 * Tonsil *_R_*(MD_50_)
+372.00 * Heschl *_L_*(FA_90_)	−1602.36 * Biventral *_L_*(FA_10_)	−753.08 * MiddleFrontal *_R_*(MD_90_)
+2118.39 * Postcentral *_R_*(MD_50_)	−1431.37 * SuperiorOccipital *_L_*(MD_50_)	+2573.81 * Cuneus *_R_*(FA_50_)
+486.64 * SuperiorFrontal, MedialOrbital *_R_*(MD_90_)	−1853.35 * Thalamus *_L_*(FA_50_)	+1547.72 * InferiorFrontal, Triangular *_R_*(FA_50_)
+298.36 * Alae *_R_*(MD_10_)	−1597.47	
**PDQ39SI** =		
+370.38 * Quadrangular *_R_*(FA_10_)	+208.71 * RolandicOperculum *_R_*(FA_50_)	+44.90 * SuperiorTemporal *_R_*(MD_90_)
+125.00 * Cingulum, Anterior *_R_*(MD_10_)	−10.77 * Biventral *_L_*(MD_90_)	+46.65 * Cingulum, Posterior *_R_*(MD_10_)
+10.55 * Lingula(MD_50_)	−107.68 * MiddleOccipital *_L_*(MD_10_)	+78.75 * Biventral *_R_*(FA_10_)
+73.82 * ParacentralLobule *_L_*(MD_10_)	−28.89 * Caudate *_L_*(MD_10_)	−71.872 * Insula *_R_*(MD_10_)
+279.08 * SuperiorFrontal, Medial *_R_*(FA_10_)	−211.64	
**MHY** =		
+8.02 * Nodule(FA_50_)	+10.74 * Angular *_L_*(MD_50_)	+1.99 * RolandicOperculum *_L_*(FA_90_)
−2.70 * ParacentralLobule *_R_*(FA_90_)	+9.83 * Cuneus *_L_*(FA_50_)	+0.55 * Lingula(MD_50_)
+1.37 * Heschl *_L_*(MD_10_)	−6.39 * Insula *_R_*(MD_10_)	+2.22 * Amygdala *_R_*(MD_10_)
+0.56 * Caudate *_L_*(MD_90_)	−1.43 * SuperiorFrontal, Orbital *_R_*(MD_90_)	+9.87 * FoliumTuber(FA_10_)
−5.85 * Rectus *_R_*(FA_10_)	−9.14	

LEDD: levodopa equivalent daily dose; PDQ39SI: Summary Index of 39-item PD Questionnaire; MHY: Modified Hoehn and Yahr staging; MD, mean diffusivity; FA, fractional anisotropy; *R*, right hemisphere; *L*, left hemisphere; 90, 90th percentile; 50, 50th percentile; 10, 10th percentile; * indicated multiplication.
